# Epac1^–/–^ and Epac2^–/–^ mice exhibit deficient epithelial Na^+^ channel regulation and impaired urinary Na^+^ conservation

**DOI:** 10.1172/jci.insight.145653

**Published:** 2022-02-08

**Authors:** Viktor N. Tomilin, Kyrylo Pyrshev, Anna Stavniichuk, Naghmeh Hassanzadeh Khayyat, Guohui Ren, Oleg Zaika, Sherif Khedr, Alexander Staruschenko, Fang C. Mei, Xiaodong Cheng, Oleh Pochynyuk

**Affiliations:** 1 Department of Integrative Biology and Pharmacology, The University of Texas Health Science Center at Houston, Houston, Texas, USA.; 2 Department of Physiology, Medical College of Wisconsin, Milwaukee, Wisconsin, USA.; 3 Department of Physiology, Faculty of Medicine, Ain-Shams University, Cairo, Egypt.; 4 Department of Molecular Pharmacology and Physiology, University of South Florida, Tampa, Florida, USA.

**Keywords:** Nephrology, Epithelial transport of ions and water, Ion channels, Sodium channels

## Abstract

Exchange proteins directly activated by cAMP (Epacs) are abundantly expressed in the renal tubules. We used genetic and pharmacological tools in combination with balance, electrophysiological, and biochemical approaches to examine the role of Epac1 and Epac2 in renal sodium handling. We demonstrate that Epac1^–/–^ and Epac2^–/–^ mice exhibit a delayed anti-natriuresis to dietary sodium restriction despite augmented aldosterone levels. This was associated with a significantly lower response to the epithelial Na^+^ channel (ENaC) blocker amiloride, reduced ENaC activity in split-opened collecting ducts, and defective posttranslational processing of α and γENaC subunits in the KO mice fed with a Na^+^-deficient diet. Concomitant deletion of both isoforms led to a marginally greater natriuresis but further increased aldosterone levels. Epac2 blocker ESI-05 and Epac1&2 blocker ESI-09 decreased ENaC activity in Epac WT mice kept on the Na^+^-deficient diet but not on the regular diet. ESI-09 injections led to natriuresis in Epac WT mice on the Na^+^-deficient diet, which was caused by ENaC inhibition. In summary, our results demonstrate similar but nonredundant actions of Epac1 and Epac2 in stimulation of ENaC activity during variations in dietary salt intake. We speculate that inhibition of Epac signaling could be instrumental in treatment of hypertensive states associated with ENaC overactivation.

## Graphical abstract

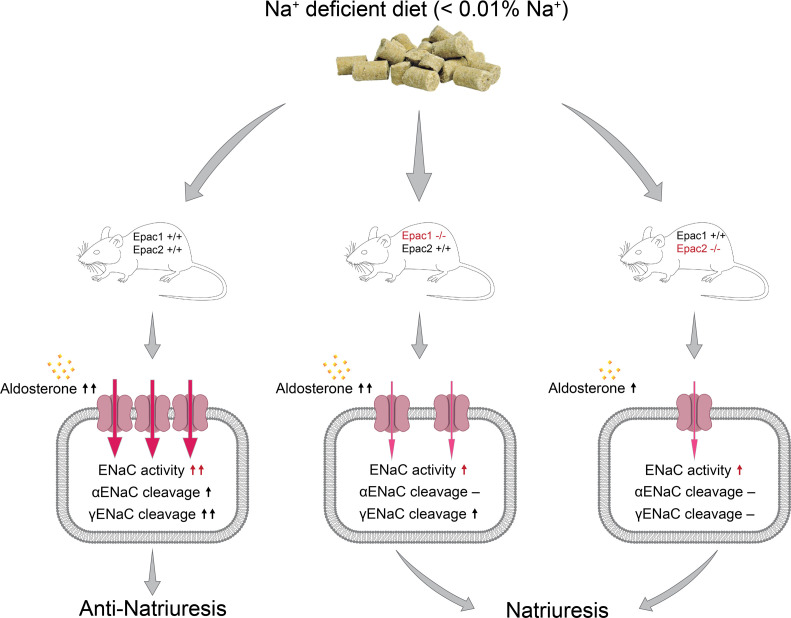

## Introduction

The kidneys are the master regulators of systemic homeostasis, extracellular fluid volume, and blood pressure by controlling urinary excretion of water and electrolytes (1). Dietary Na^+^ restriction/load stimulates/inhibits Na^+^ reabsorption in the renal collecting duct, respectively, in order to maintain sodium balance and normal blood pressure (2, 3). At the molecular level, activity of the epithelial Na^+^ channel (ENaC) mediates electrogenic Na^+^ reabsorption in the collecting duct principal cells, which also creates a driving force for K^+^ secretion via the ROMK (K_ir_1.1) potassium channel (2). ENaC is a highly selective Na^+^ channel composed of α, β, and γ subunits in 1:1:1 stoichiometry (4). Mutations in genes encoding ENaC subunits result in monogenic forms of hypertension (gain-of-function) and hypotension (loss-of-function) in humans (5). Furthermore, many ENaC polymorphisms have been associated with the development of salt-sensitive hypertension in different ethnic populations (6–8).

Several endocrine factors, such as aldosterone, Angiotensin II, and Arginine Vasopressin (AVP), stimulate ENaC-mediated Na^+^ reabsorption in the collecting duct to decrease urinary sodium excretion in response to hypovolemia and dietary Na^+^ restriction (9–12). This manifests as increased ENaC synthesis, enhanced trafficking to the apical plasma membrane, channel activation due to proteolytic cleavage of α and γ subunits, and augmented gating (3, 4). Of interest, endocrine signals commonly lead to elevations in cAMP levels in the collecting duct principal cells (13–15). In turn, high cAMP could serve as a critical second messenger to augment ENaC-mediated Na^+^ reabsorption. Consistently, activation of the cAMP pathway promotes ENaC trafficking to the apical membrane (16, 17) and increases ENaC open probability in cultured and native collecting duct cells (12). However, much less is known about cAMP-mediated downstream pathways in regulation of ENaC functions in the collecting duct. Exchange proteins directly activated by cAMP (Epacs) are guanine nucleotide exchange factors for Ras-like small GTPases, Rap1 and Rap2 (18, 19). Binding of cAMP to the cyclic nucleotide binding (CNB) domain of Epac leads to conformational changes and its activation (20, 21). Importantly, the cAMP–Epac cascade often operates independently from the classical cAMP–protein kinase A (PKA) signaling (22). Epac is recognized as playing a role in a variety of cellular processes, and augmented Epac signaling has been reported in many diseases, including cancer, diabetic nephropathy, and neurological and vascular disorders (23–28).

The known Epac isoforms, Epac1 and Epac2, are abundantly expressed in the renal tubule cells of rodents and humans where they are likely involved in modulation of water-electrolyte transport (29). Indeed, we recently demonstrated that Epac1^–/–^ and Epac2^–/–^ mice developed polyuria and impaired urinary concentration ability in response to water deprivation driven, in part, by decreased NHE-3 (*SLC9A3*) expression in the proximal tubule (30). However, little is known about molecular targets and significance of Epac signaling in the distal nephron segments, including the collecting duct, which is involved in the fine-tuning of systemic Na^+^ balance and setting salt-sensitivity of blood pressure.

In this study, we combined a variety of systemic balance and electrophysiological, biochemical, genetic, and pharmacological approaches to address the following research questions: 1) Does Epac signaling play a role in renal conservation of sodium and specifically in salt-sensitive changes in ENaC activity and expression in the collecting duct?; 2) Are both Epac1 and Epac2 necessary for ENaC functions?; and 3) Does pharmacological Epac inhibition produce natriuresis by blocking ENaC activity?

## Results

### Epac isoform-deficient mice exhibit impaired adaptation to dietary salt restriction.

**Figure 1:**
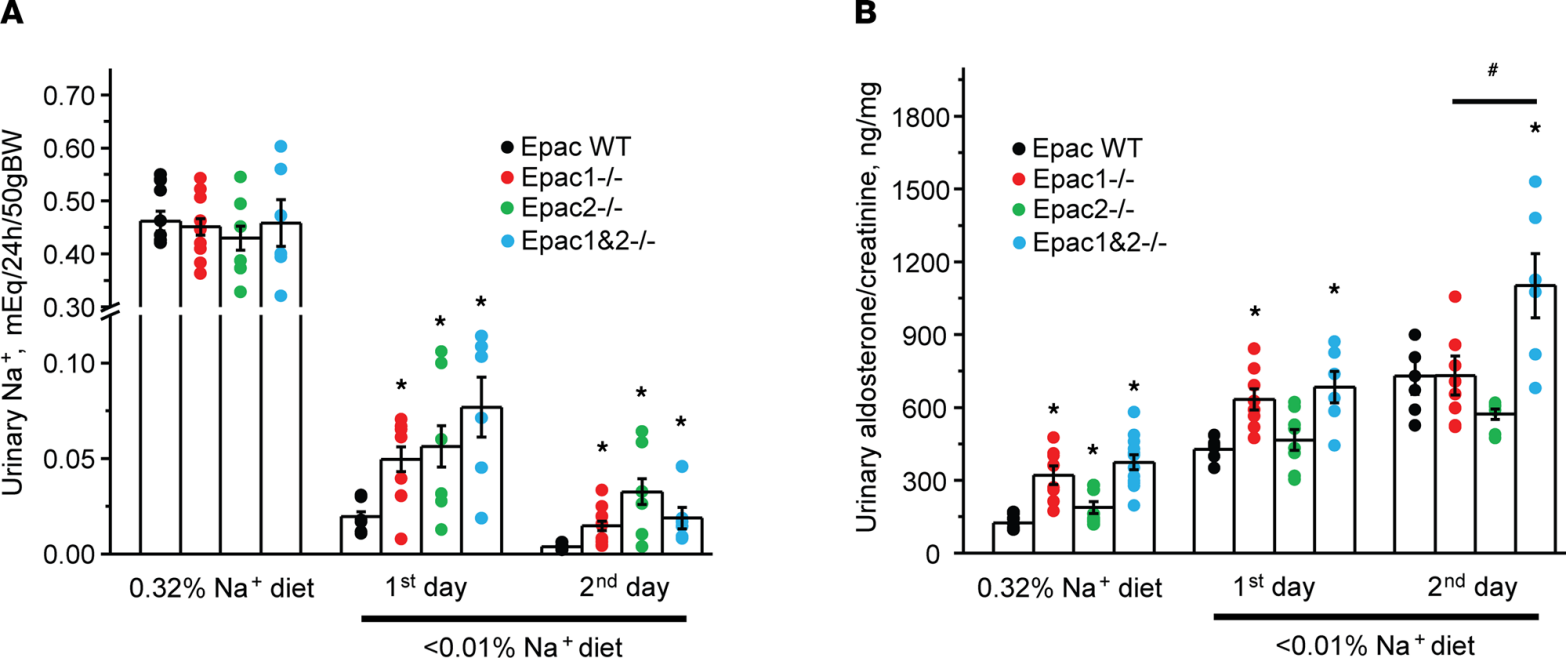
Epac1^–/–^, Epac2^–/–^, and Epac1&2^–/–^ mice exhibit impaired adaptation to dietary sodium restriction. The summary graphs showing a comparison of 24-hour urinary Na^+^ levels (**A**) and urinary aldosterone to creatinine ratio (**B**) in Epac WT, Epac1^–/–^, Epac2^–/–^, and Epac1&2^–/–^ mice maintained on a regular (0.32% Na^+^) diet and in response to a sodium-deficient diet (< 0.01% Na^+^) for 1 or 2 days. Each dot represents measurement from individual animals. *P < 0.05 versus the respective values in Epac WT mice; ^#^*P* < 0.05 versus Epac1^–/–^ and Epac2^–/–^. A 1-way ANOVA with post hoc Tukey test was used.

We first used systemic balance studies in metabolic cages to examine the role of Epac signaling in renal sodium handling during variations in dietary salt intake. Upon acclimation, the 24-hour urinary Na^+^ levels were comparable in Epac WT, Epac1^–/–^, and Epac2^–/–^ mice kept on a regular (0.32% Na^+^) diet (Figure 1A). Switching to a sodium-deficient diet (< 0.01% Na^+^) produced a rapid decrease in urinary Na^+^ levels in Epac WT mice within 2 days (Figure 1A). In contrast, both KOs excreted significantly larger amounts of Na^+^ in urine during the transition period, thus exhibiting an impaired response to dietary sodium restriction (Figure 1A). At the same time, there were no measurable differences in plasma Na^+^ and K^+^ concentrations (Supplemental Figure 1; supplemental material available online with this article; https://doi.org/10.1172/jci.insight.145653DS1) between Epac WT, Epac1^–/–^, and Epac2^–/–^ mice kept on the regular and sodium-deficient (for 2 days) diets. Importantly, urinary aldosterone levels were significantly elevated (more in Epac1^–/–^ and less in Epac2^–/–^) when mice were fed a regular diet (Figure 1B). Dietary sodium restriction further augmented aldosterone levels in Epac1^–/–^ and Epac2^–/–^ mice, suggesting that the observed urinary salt wasting is likely a result of diminished Na^+^ reabsorption in the nephron and not due to the deficient aldosterone synthesis/secretion. However, the magnitude of the response was somewhat lower in Epac2^–/–^ (Figure 1B). This is consistent with greater urinary Na^+^ levels in mice lacking this isoform.

Since deletion of either Epac isoform compromises adaptation to dietary sodium restriction, we next examined Na^+^ balance in mice lacking both Epac isoforms (Epac1&2^–/–^, Supplemental Figure 2). The 24-hour urinary Na^+^ levels were comparable to Epac WT and single Epac isoform KOs on the regular (0.32% Na^+^) diet (Figure 1A), while the aldosterone levels were significantly elevated (Figure 1B). Dietary Na^+^ restriction induced modestly increased urinary Na^+^ compared with Epac1^–/–^ and Epac2^–/–^ mice on the first, but not on the second day (Figure 1A). Importantly, aldosterone levels were further significantly elevated in Epac1&2^–/–^ mice when compared with the single isoform KOs on the second day (Figure 1B). We concluded that the lack of additive natriuretic effect is largely mitigated by higher aldosterone levels in Epac1&2^–/–^ mice. Overall, the results in Figure 1 demonstrate that mice with deletion of a single or both Epac isoforms exhibit an impaired anti-natriuretic response.

### Diminished amiloride-induced natriuresis in Epac isoform-deficient mice.

**Figure 2:**
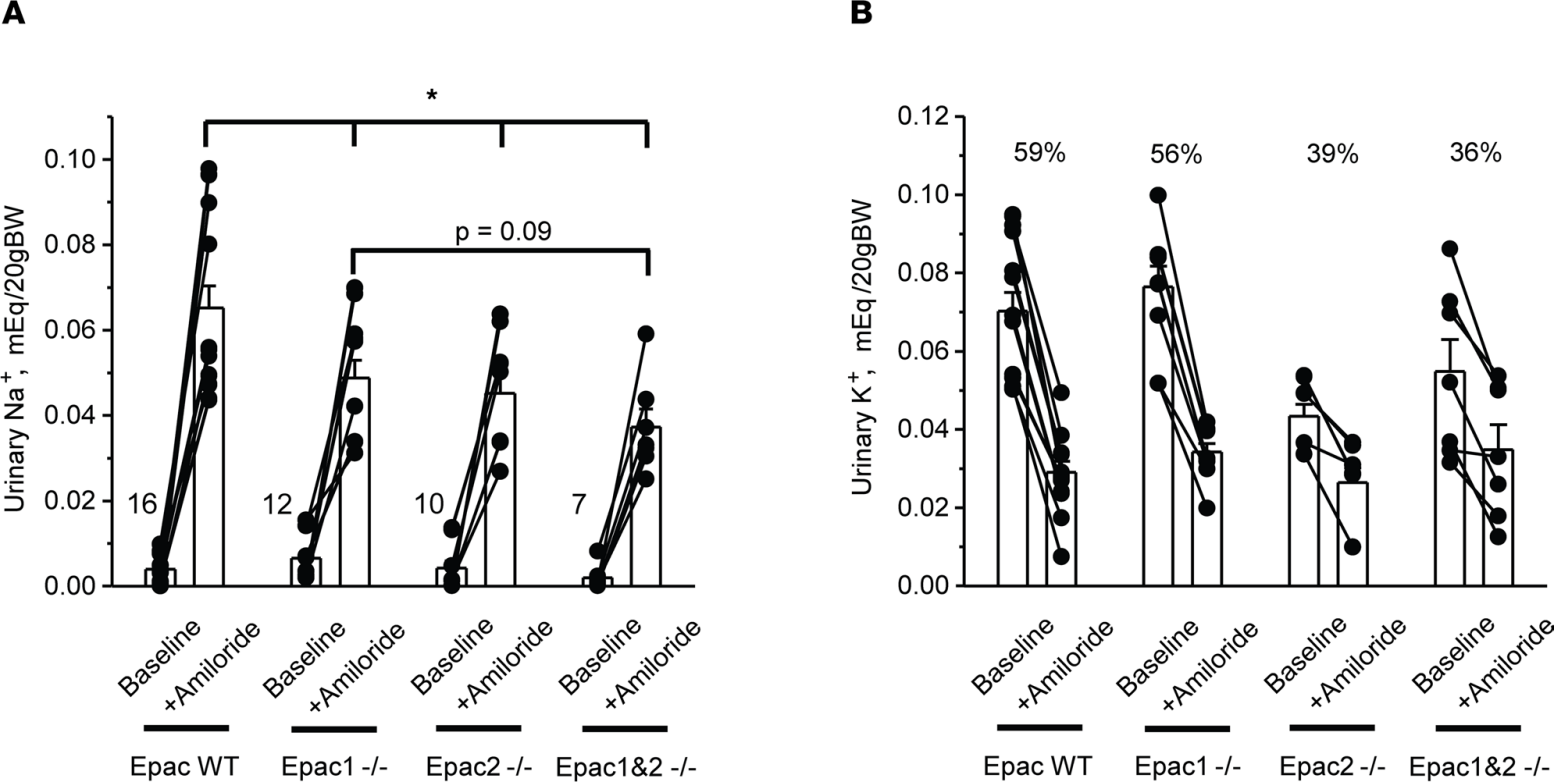
Reduced natriuresis to the ENaC blocker amiloride in Epac1^–/–^, Epac2^–/–^, and Epac1&2^–/–^ mice. Summary graphs comparing urinary Na^+^ levels (**A**) and K^+^ levels (**B**) in Epac WT, Epac1^–/–^, Epac2^–/–^, and Epac1&2^–/–^ mice kept on Na^+^-deficient diet for 7 days at the baseline and following i.p. injection of amiloride (2 mg/kgBW). Measurements from the same animal are connected with lines. In both cases, urine samples were collected over a 6-hour period. Relative reductions (in %) in urinary K^+^ levels are shown for each tested group. *P < 0.05 versus Epac WT mice + Amiloride; 1-way ANOVA with post hoc Tukey test was used.

The salt wasting phenotype in Epac1^–/–^ and Epac2^–/–^ mice (Figure 1) indicates either diminished ENaC-dependent Na^+^ reabsorption in the collecting duct due to resistance to aldosterone or a defect in upstream nephron segments with compensatory stimulation of ENaC by aldosterone. Thus, we next assessed natriuretic response to injection of the ENaC blocker, amiloride (2 mg/kgBW) in Epac WT, Epac1^–/–^, Epac2^–/–^, and Epac1&2^–/–^ mice fed a Na^+^-deficient diet (< 0.01% Na^+^) for 7 days. As summarized in Figure 2A, Epac1^–/–^ and Epac2^–/–^ mice exhibited significantly blunted amiloride-induced elevations in urinary Na^+^ levels indicative of decreased ENaC activity. Furthermore, the natriuretic effect of amiloride had a trend of being further reduced in Epac1&2^–/–^ mice (*P* = 0.09) compared with that observed in Epac1^–/–^ (Figure 2A). During the baseline period, urinary aldosterone/creatinine ratios on day 7 were: 730 ± 116 ng/mg (Epac WT); 891 ± 139 ng/mg (Epac1^–/–^); 738 ± 53 ng/mg (Epac2^–/–^); and 1112 ± 150 ng/mg (Epac1&2^–/–^). These values are consistent with those reported in Figure 1B on day 2 of the sodium-deficient diet, with Epac1&2^–/–^ having the highest levels of aldosterone. To ensure that the amiloride-induced natriuresis represented chiefly inhibition of ENaC-mediated Na^+^ reabsorption in the collecting duct principal cells, we also monitored changes in urinary K^+^ levels (Figure 2B). Amiloride significantly decreased excretion of K^+^ with urine in all tested groups. The magnitude was the highest in Epac WT and the lowest in Epac1&2^–/–^, being inversely related to the respective natriuresis, as anticipated (Figure 2B).

### Epac1 and Epac2 deletion decreases single-channel ENaC activity in a salt-dependent manner.

**Figure 3:**
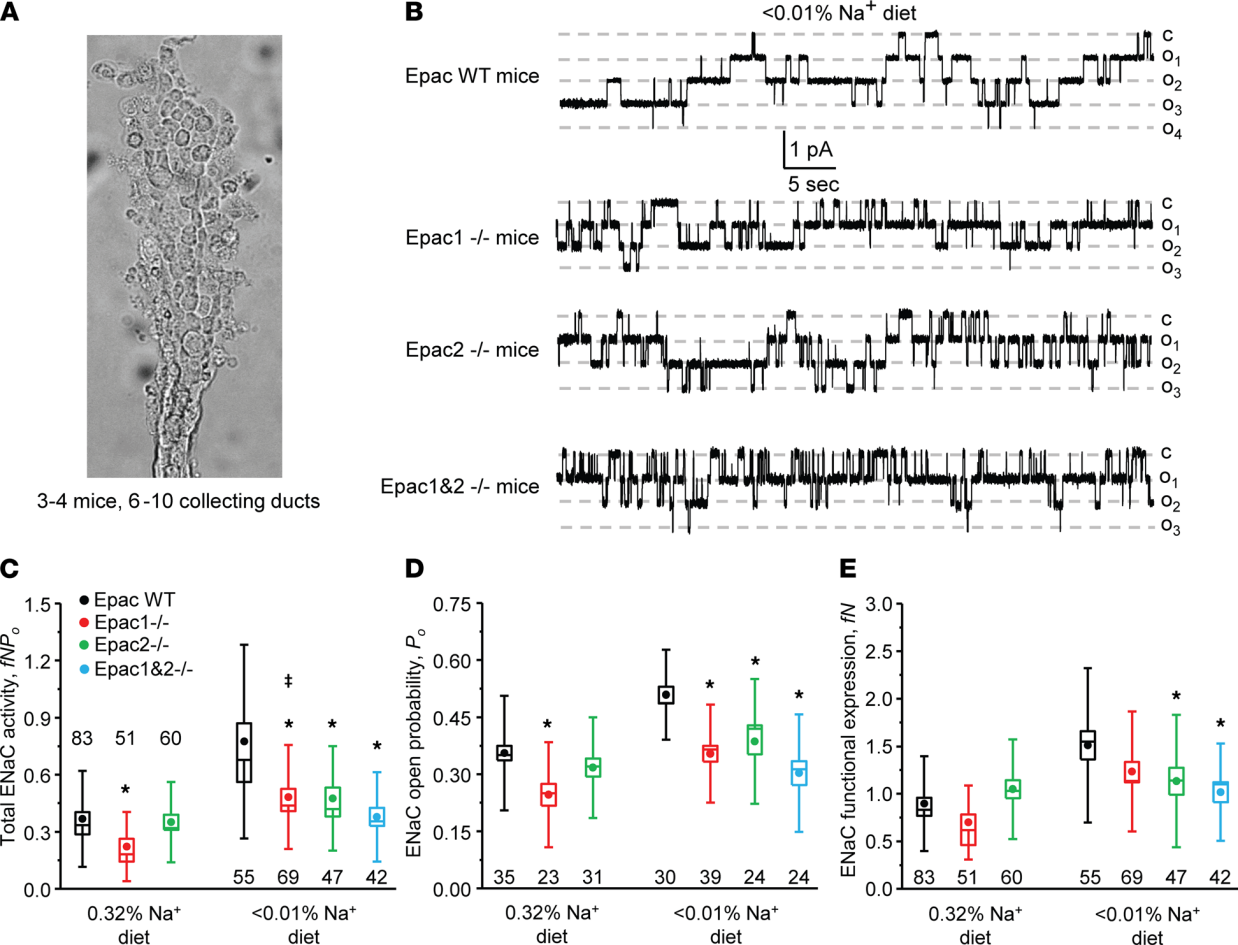
Deletion of Epac1, Epac2, and Epac1&2 decreases single-channel ENaC activity in the collecting duct. (**A**) A representative image of a split-opened collecting duct suitable for patch clamp assessment of ENaC activity. On average, 6–10 individual collecting ducts from 3–4 different mice were used for each tested condition. (**B**) Representative current traces of ENaC activity in split-opened collecting ducts isolated from Epac WT, Epac1^–/–^, Epac2^–/–^, and Epac1&2^–/–^ mice kept on sodium-deficient diet (< 0.01% Na^+^) for 1 week. The patches were held at a test potential of V_h_ = −V_p_ = −60 mV. Inward currents are downward. Dashed lines indicate the respective current state with *o_i_* indicating the number of simultaneously opened channels and *c* denoting the closed nonconducting state. Summary whisker graphs of total ENaC activity, *fNP_o_* (**C**); ENaC open probability, *P_o_* (**D**); and number of active channels *fN* (**E**) in Epac WT (black), Epac1^–/–^ (red), Epac2^–/–^ (green), and Epac1&2^–/–^ (blue) mice kept on regular (0.32% Na^+^) and sodium-deficient (< 0.01% Na^+^) diets. Means are shown with dots, medians are highlighted with lines, bars represent standard error, and whiskers define standard deviation. *P < 0.05 versus respective Epac WT; ^ǂ^*P* < 0.05 versus Epac1^–/–^ mice on regular Na^+^ diet. A 1-way ANOVA with post hoc Tukey test was used.

We next directly monitored single-channel ENaC activity with patch clamp electrophysiology in freshly isolated split-opened collecting ducts (Figure 3A) of Epac WT, Epac1^–/–^, Epac2^–/–^, and Epac1&2^–/–^ mice fed sodium-deficient (< 0.01% Na^+^) and regular (0.32% Na^+^) diets. As shown on the representative current traces (Figure 3B) and the respective summary graph (Figure 3C), total ENaC activity (*fNP_o_*) was significantly lower in Epac1^–/–^ and Epac2^–/–^ mice compared with Epac WT mice on a Na^+^-deficient diet. Concomitant deletion of Epac1 and Epac2 had a tendency to further reduce total ENaC activity, but this did not attain a significant difference. The analysis of patch clamp data revealed a significantly lower channel open probability (*P_o_*) in all KO strains (Figure 3D) and a significantly decreased average number of active channels in a patch (*fN*) in Epac2^–/–^ and Epac1&2^–/–^ mice compared with Epac WT. We also noticed a similar tendency for decreased active channel numbers in Epac1^–/–^ animals, but this did not reach a significant difference (Figure 3E). Furthermore, we found a moderately reduced total ENaC activity only in Epac1^–/–^ mice on the regular Na^+^ intake (Figure 3C) due to a lower open probability (Figure 3D), whereas channel activity was comparable in Epac WT and Epac2^–/–^ mice in this condition (Figure 3, C–E). Interestingly, deletion of Epac1 did not abolish upregulation of ENaC activity by dietary salt restriction, while the total activity was significantly lower compared with Epac WT under both conditions (Figure 3C). In contrast, total ENaC activity was not significantly different in Epac2^–/–^ mice in regular and salt-deficient conditions (Figure 3C). In summary, our patch clamp studies show the critical contribution of the Epac signaling cascade in controlling ENaC activity in the collecting duct. Specifically, Epac1 deletion reduced ENaC activity on both regular and sodium-deficient diets and Epac2 ablation impaired the upregulation of the channel activity by dietary salt restriction. ENaC activity was the lowest upon concomitant deletion of Epac1 and Epac2 but was not significantly different from that in Epac1^–/–^ and Epac2^–/–^ mice kept on a sodium-deficient diet.

### Epac1 and Epac2 control posttranslational modifications of α and γENaC.

**Figure 4:**
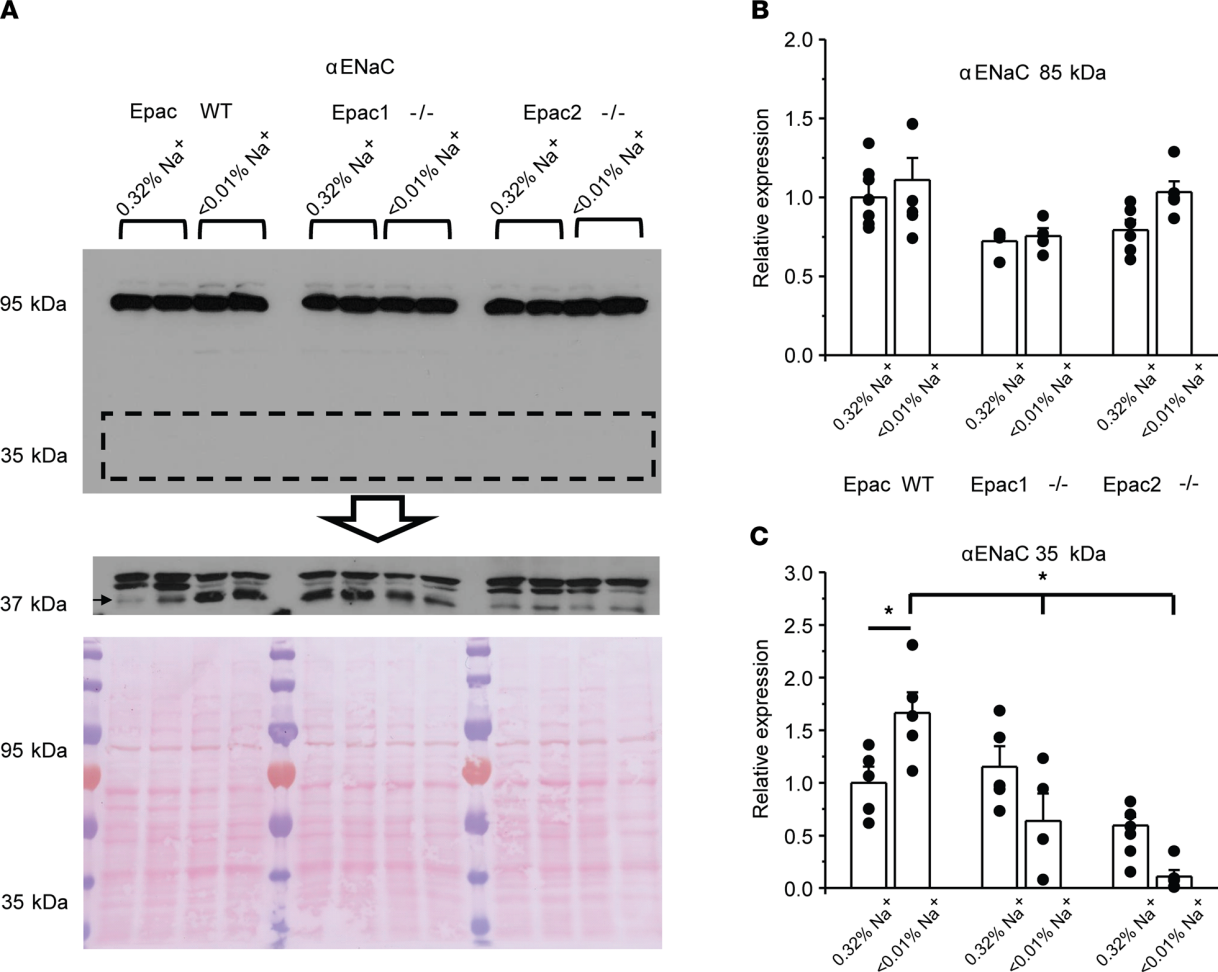
Defective renal αENaC processing in mice lacking Epac isoforms. (**A**) Representative Western blot from whole kidney lysates of Epac WT, Epac1^–/–^, and Epac2^–/–^ mice kept on regular (0.32% Na^+^) and sodium-deficient (< 0.01% Na^+^) diets probed with anti-αENaC antibodies using low sensitivity (top panel) and high sensitivity (middle panel) chemiluminescence kits. Signal around 95 kDa corresponds to full-length αENaC and signal around 37 kDa reflects cleaved αENaC form (showed with an arrow). The Ponceau red staining of the same nitrocellulose membrane demonstrating equal protein loading is shown on the bottom panel. (**B** and **C**) Summary graphs comparing full-length and cleaved forms of αENaC expression in Epac WT, Epac1^–/–^, and Epac2^–/–^ mice under conditions in **A**. Each measurement represents an individual mouse. The intensity values were normalized to the total signal of the respective lines in Ponceau red staining. *P < 0.05 between experimental groups shown with a line on the top; 1-way ANOVA with post hoc Tukey test was used.

We next examined whether diminished ENaC activity is associated with decreased expression and/or defective cleavage of α and γENaC subunits in mice lacking Epac isoforms. As shown in the representative Western blot and summary graph (Figure 4, A and B), the full-length (~95 kDa) αENaC levels were comparable in whole kidney lysates of Epac WT, Epac1^–/–^, and Epac2^–/–^ mice fed both regular (0.32% Na^+^) and sodium-deficient (< 0.01% Na^+^) diets. Abundant published evidence suggests an important role of αENaC cleavage by proteases, such as furin, by producing an approximately 35 kDa N-terminus fragment in stimulation of channel activity during variations in dietary salt intake (as reviewed in ref. 4). Using a high-sensitivity chemiluminescence kit on the same nitrocellulose membrane, 3 bands of a similar size were detected by antibodies against αENaC (Figure 4A). However, only the lowest molecular weight band (shown with arrow) was present in whole cell lysates from mpkCCD_c14_ cells (Supplemental Figure 3), a generally accepted model of principal cell of the collecting duct (31). Thus, we considered that this is the cleaved form of αENaC and the upper 2 bands are not specific. As expected, we observed a notable increase in abundance of the cleaved αENaC in Epac WT mice fed a sodium-deficient diet compared with the control (Figure 4C). In contrast, we did not detect such upregulation in kidney lysates from Epac1^–/–^ and Epac2^–/–^ mice under the dietary sodium restriction condition (Figure 4C).

**Figure 5:**
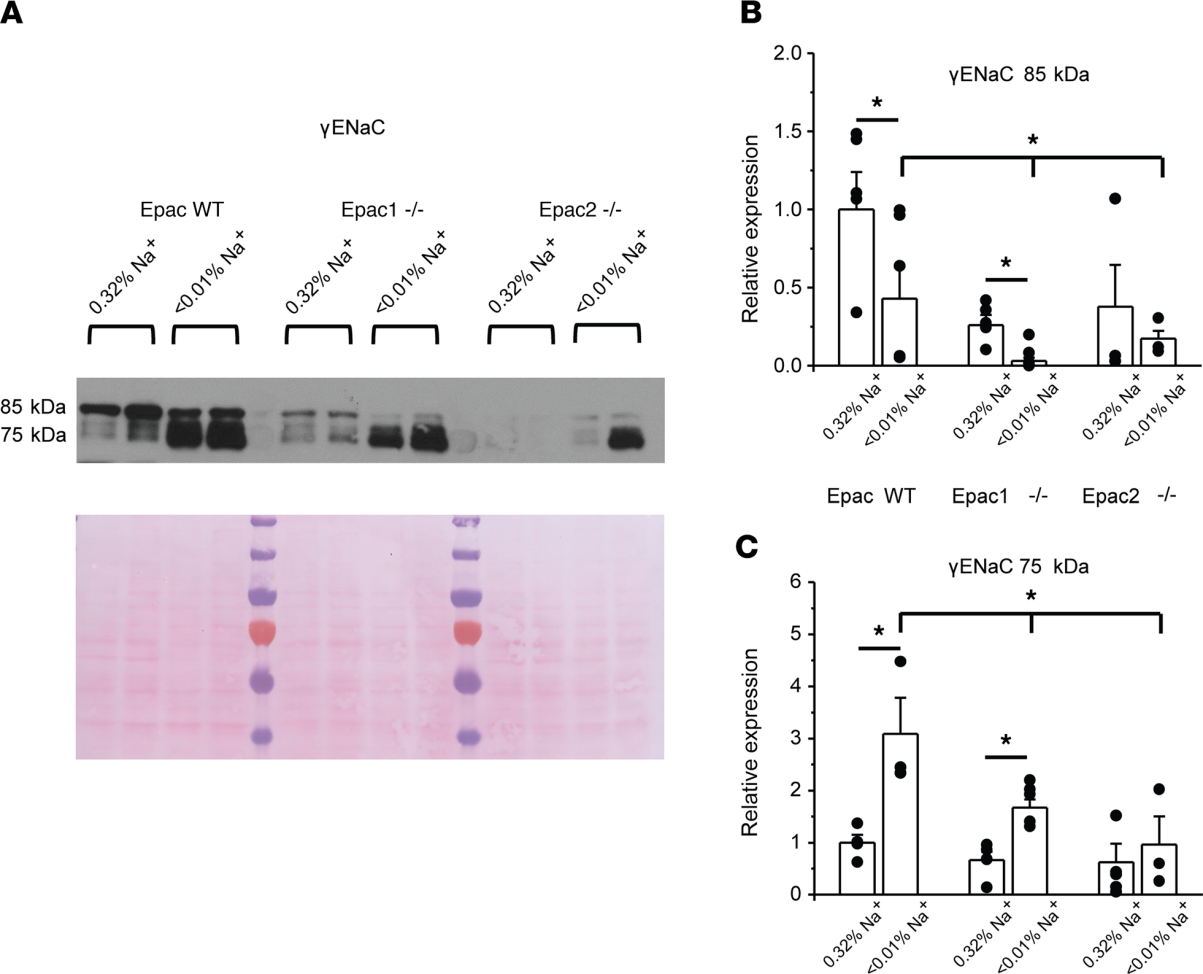
Deletion of Epac isoforms decreases γENaC cleavage. (**A**) Representative Western blot from whole kidney lysates of Epac WT, Epac1^–/–^, and Epac2^–/–^ mice kept on regular (0.32% Na^+^) and sodium-deficient (< 0.01% Na^+^) diets probed with anti-γENaC antibodies. Signal around 85 kDa corresponds to full-length (low active) γENaC and signal around 75 kDa reflects cleaved active γENaC form. (**B** and **C**) Summary graphs comparing the abundance of full-length and cleaved forms of γENaC in Epac WT, Epac1^–/–^, and Epac2^–/–^ mice under conditions in **A**. Each measurement represents an individual mouse. The intensity values were normalized to the total signal of the respective lines in Ponceau red staining. *P < 0.05 between experimental groups showed with a line on the top; 1-way ANOVA with post hoc Tukey test was used.

While βENaC does not seem to be regulated by variations in dietary salt intake (32), cleavage of the full-length γENaC (~85 kDa) to yield an approximately 75 kDa fragment is known to be critical for activation of the channel (4). Consistently, we observed a dramatic increase in the ratio of active (75 kDa) to inactive (85 kDa) forms of γENaC in Epac WT mice fed a Na^+^-deficient diet (Figure 5A). Figure 5B shows that the reduction of the full-length γENaC (~85 kDa) is blunted in Epac1^–/–^ and Epac2^–/–^ mice. Furthermore, the upregulation of the active (75 kDa) γENaC form is diminished in Epac1^–/–^ and is abolished in Epac2^–/–^ (Figure 5C). Altogether, our Western blot experiments show defective regulation of α and γ ENaC subunits in response to dietary salt restriction in Epac1^–/–^ and Epac2^–/–^ mice (Figures 4 and 5). This is consistent with the diminished ENaC activity in both mutants, as was measured with patch clamp electrophysiology (Figure 3).

### Acute inhibition of Epac signaling decreases ENaC activity in the collecting duct.

**Figure 6:**
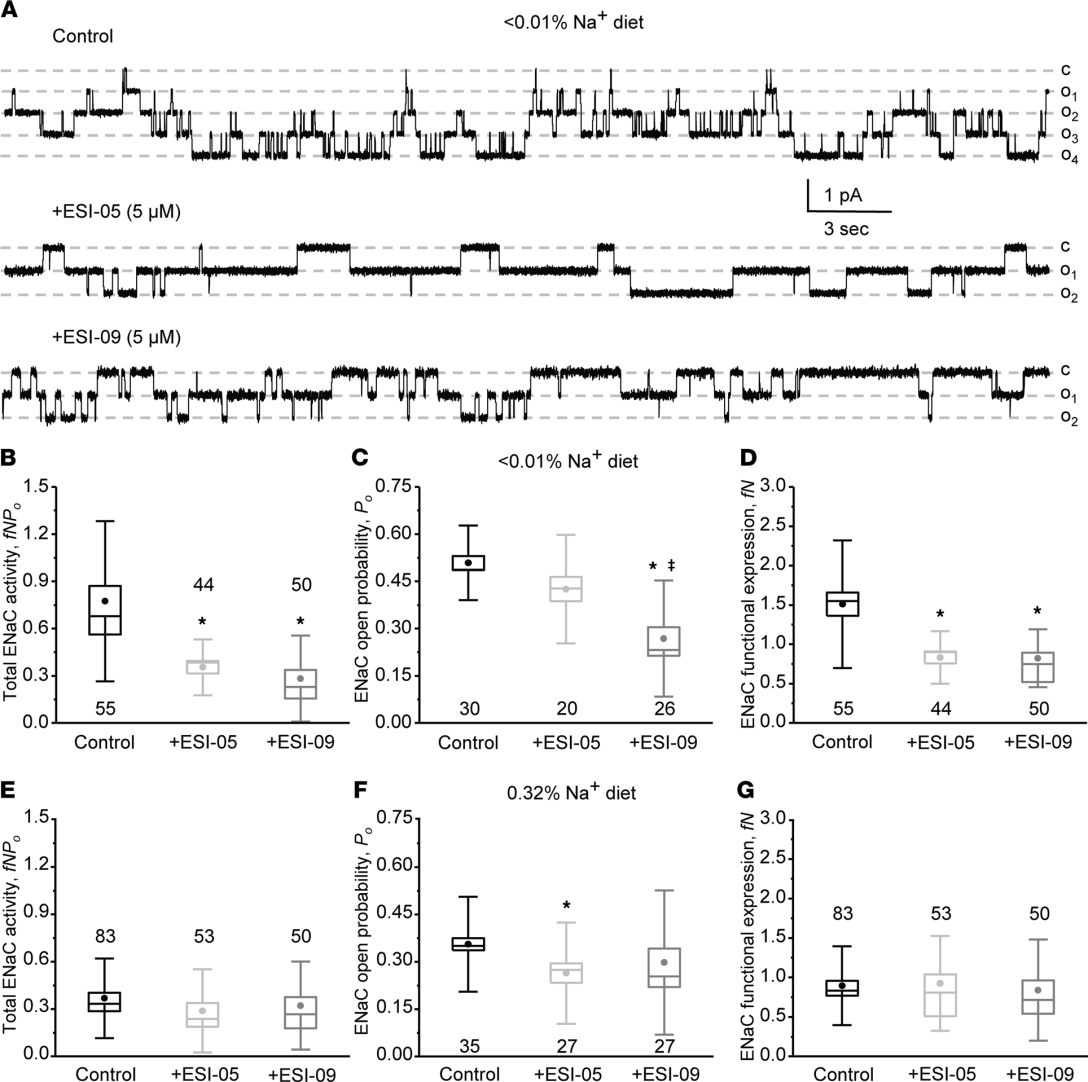
Acute inhibition of Epac signaling decreases ENaC activity in Epac WT mice kept on a sodium-deficient diet. (**A**) Representative current traces of ENaC activity in split-opened collecting ducts in the control (top), pretreated with selective Epac2 inhibitor ESI-05 (5 μM) and combined Epac1&2 blocker ESI-09 (5 μM) for 15 minutes. The collecting ducts were isolated from Epac WT mice kept on a sodium-deficient (< 0.01% Na^+^) diet for 1 week. The patches were held at a test potential of V_h_ = −V_p_ = −60 mV. Inward currents are downward. Dashed lines indicate the respective current state with *o_i_* indicating the number of simultaneously opened channels and *c* denoting the closed nonconducting state. Summary whisker graphs of total ENaC activity, *fNP_o_* (**B**); ENaC open probability, *P_o_* (**C**); and number of active channels *fN* (**D**) in Epac WT mice kept on sodium-deficient (< 0.01% Na^+^) diet in the control and after pretreatment with ESI-05 and ESI-09, as described above. Means are shown with dots, medians are highlighted with lines, bars represent standard error, and whiskers define standard deviation. *P < 0.05 versus respective Control; ǂ*P* < 0.05 versus + ESI-05. One-way ANOVA with post hoc Tukey test was used. Summary whisker graphs of total ENaC activity, *fNP_o_* (**E**); ENaC open probability, *P_o_* (**F**); and number of active channels *fN* (**G**) in Epac WT mice kept on regular sodium (0.32% Na^+^) diet in the control and pretreated ESI-05 and ESI-09, as described above. *P < 0.05 versus respective Control.

Our current evidence supports a critical role of the Epac signaling cascade in stimulation of ENaC activity by dietary Na^+^ restriction. Thus, we next explored the possibility of modulating ENaC activity with Epac-related pharmacological tools. Pretreatment with selective Epac2 blocker ESI-05 (5 μM, 15 minutes; ref. 33) markedly decreased ENaC activity in Epac WT mice kept on a sodium-deficient diet (< 0.01% Na^+^), as shown on the representative current traces (Figure 6A) and the summary graph of total ENaC activity (Figure 6B). Single channel analysis revealed that *P_o_* was only marginally affected upon ESI-05 treatment (Figure 6C), whereas the number of active channels was significantly reduced compared with the control condition (Figure 6D). Furthermore, pretreatment with combined Epac1&2 inhibitor ESI-09 (5 μM, 15 minutes; ref. 34) had a modestly greater inhibitory effect on ENaC *P_o_* compared with ESI-05 (Figure 6B). In this case, we found that both the open probability (Figure 6C) and the number of active channels per patch (Figure 6D) were significantly lower compared with control. In contrast, pretreatment with either ESI-05 or ESI-09 did not significantly reduce ENaC activity in split-opened collecting duct from mice kept on regular (0.32% Na^+^) salt intake (Figure 6E). While we detected a mildly reduced open probability in both cases (Figure 6F), there were no significant differences in the average number of active channels per patch (Figure 6G), nor in total ENaC activity (Figure 6E).

**Figure 7:**
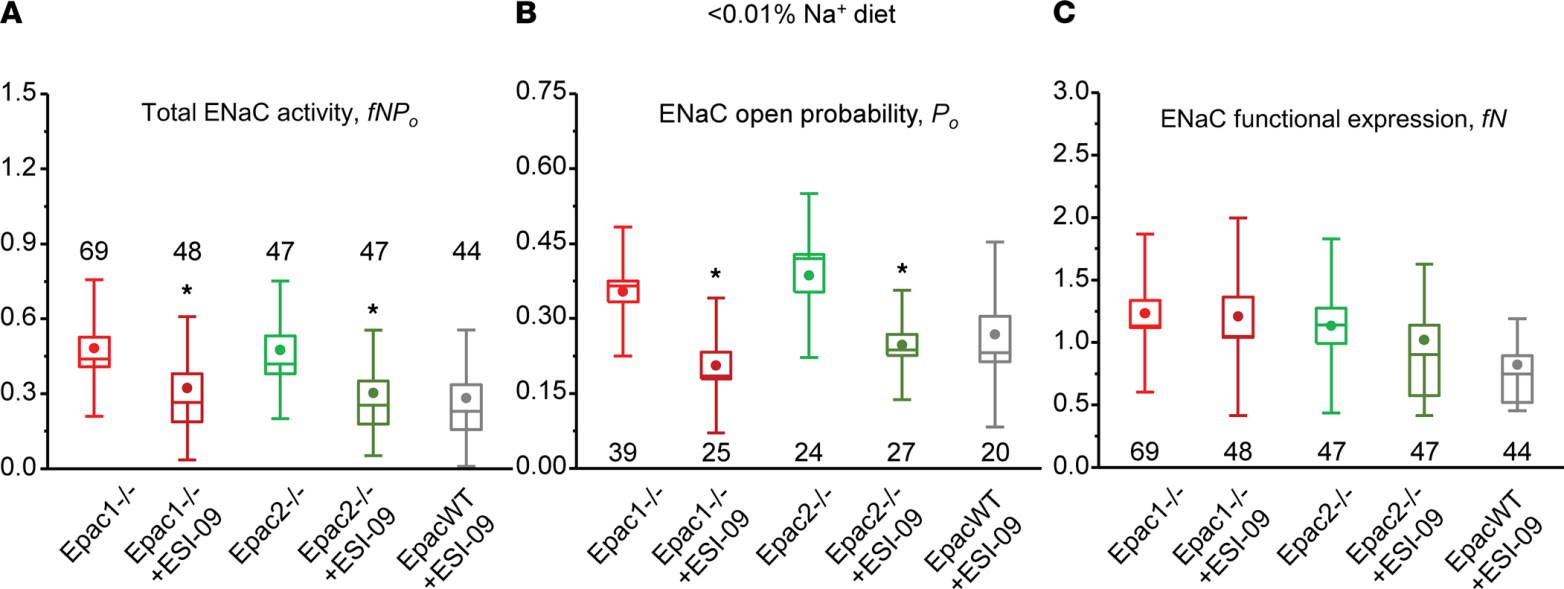
Moderate inhibitory effect of combined Epac1&2 blocker ESI-09 on ENaC activity in Epac1^–/–^ and Epac2^–/–^ mice kept on a sodium-deficient diet. (**A**) Summary whisker graphs of total ENaC activity, *fNP_o_*; (**B**) of ENaC open probability, *P_o_*; and (**C**) of number of active channels *fN* in Epac1^–/–^ and Epac2^–/–^ mice kept on sodium-deficient (< 0.01% Na^+^) diet in the control and after pretreatment with ESI-09 (5 μM) for 15 minutes. The values of ENaC activity in Epac WT mice after ESI-09 from Figure 6 are shown for comparison. Means are shown with dots, medians are highlighted with lines, bars represent standard error, and whiskers define standard deviation. *P < 0.05 versus respective Epac1^–/–^ and Epac2^–/–^ values; 1-way ANOVA with post hoc Tukey test was used.

To exclude a possibility that the Epac blockers inhibit ENaC activity in mice on a low Na^+^ diet by targeting nonspecific (i.e., Epac-independent) mechanisms, we next quantified the effects of ESI-09 in Epac isoform KO mice fed with Na^+^-deficient diet. Pretreatment with ESI-09 (5 μM, 15 minutes) significantly inhibited total ENaC activity in collecting ducts from both Epac1^–/–^ and Epac2^–/–^ mice (Figure 7A). However, the magnitudes of inhibition were much smaller compared with the effect of ESI-09 in Epac WT mice (Figure 6B). This can be explained as ESI-09–dependent inhibition of the intact counterpart Epac isoform in the respective KO. Indeed, the total ENaC activity was indistinguishable in the collecting duct cells of Epac WT, Epac1^–/–^, and Epac2^–/–^ mice after pretreatment with ESI-09 (Figure 7A). Further analysis revealed that the mild inhibitory effect of ESI-09 in Epac isoform KOs was attributed to the decreased ENaC open probability (Figure 7B), whereas the number of active channels per patch was not affected (Figure 7C).

Overall, the results from Figures 6 and 7 suggest that 1) Epac cascade directly controls ENaC activity in the collecting duct; 2) blockade of either isoform is sufficient to block ENaC activity; and 3) the cumulative blockade of Epac1 and Epac2 exhibits only a mild cumulative effect, which is consistent with the results observed for double Epac KO (Figures 3 and 4) regulation of ENaC by Epac signaling, which is augmented during dietary salt restriction.

### Systemic inhibition of Epac cascade produces natriuresis in a salt-dependent manner.

**Figure 8:**
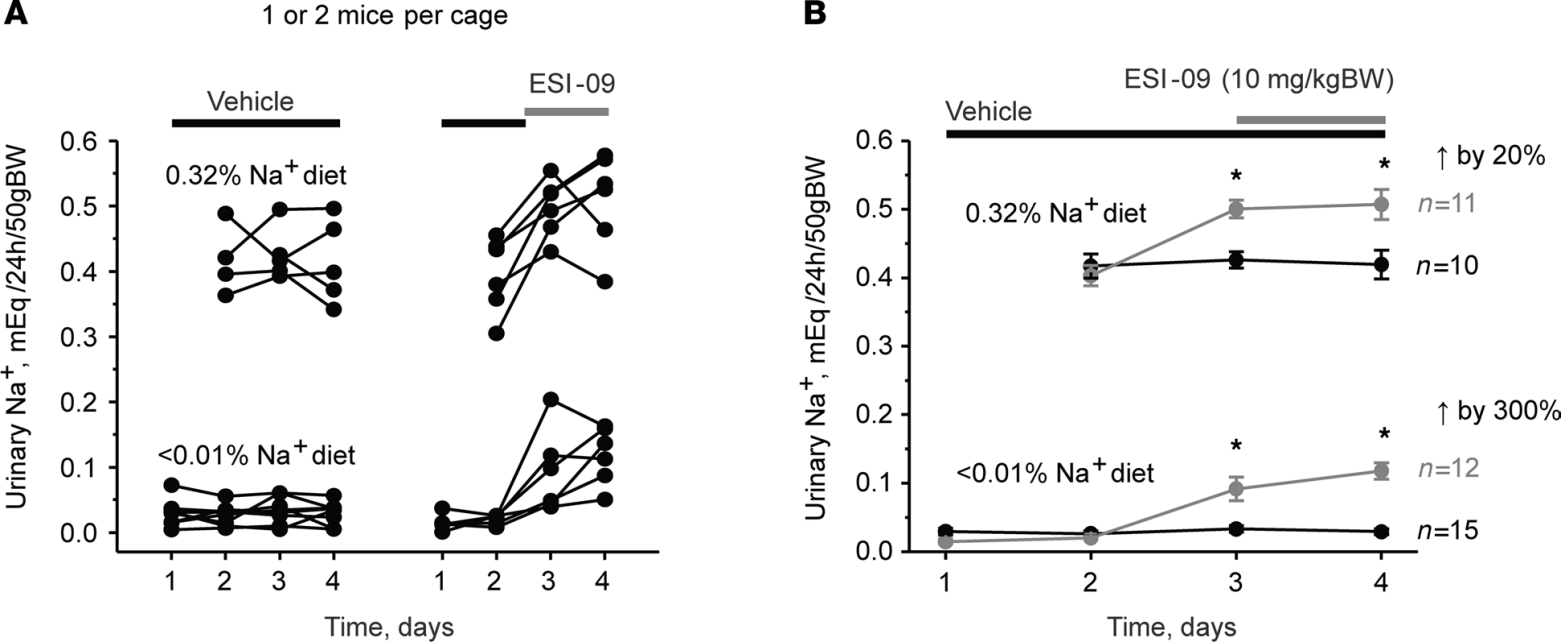
Inhibition of Epac signaling produces natriuresis in a salt-dependent manner. (**A** and **B**) The summary graphs showing a time course of changes in 24-hour urinary Na^+^ levels in individual metabolic cages per animal and the respective average values in Epac WT mice on regular (0.32% Na^+^) and sodium-deficient (< 0.01% Na^+^) diets upon daily injections of the combined Epac1&2 blocker ESI-09 (10 mg/kgBW). The number of individual mice tested for each condition is shown. *P < 0.05 versus respective vehicle condition; 1-way repeated ANOVA with post hoc Bonferroni test was used.

Our current results are consistent with the notion that both Epac1 and Epac2 are critical regulators of ENaC activity and Na^+^ reabsorption in the collecting duct, particularly during dietary sodium restriction. Thus, we next explored the consequences of systemic inhibition of Epac cascade on urinary Na^+^ levels in the control (regular Na^+^ intake; euvolemic condition) and hypovolemic (Na^+^-deficient diet) states. Daily injections of Epac1&2 inhibitor ESI-09 (10 mg/kgBW) produced a moderate but significant increase in 24-hour urinary Na^+^ by approximately 20% in Epac WT mice kept on a regular Na^+^ regimen (0.32% Na^+^) compared with vehicle injections (Figure 8, A and B). In contrast, daily ESI-09 injections increased 24-hour urinary Na^+^ approximately 3-fold compared with vehicle control in animals maintained on a sodium-deficient diet (Figure 8, A and B). This is consistent with a greater role of Epac signaling cascade in upregulation of ENaC activity during systemic volume depletion and specifically a sodium-deficient diet.

**Figure 9:**
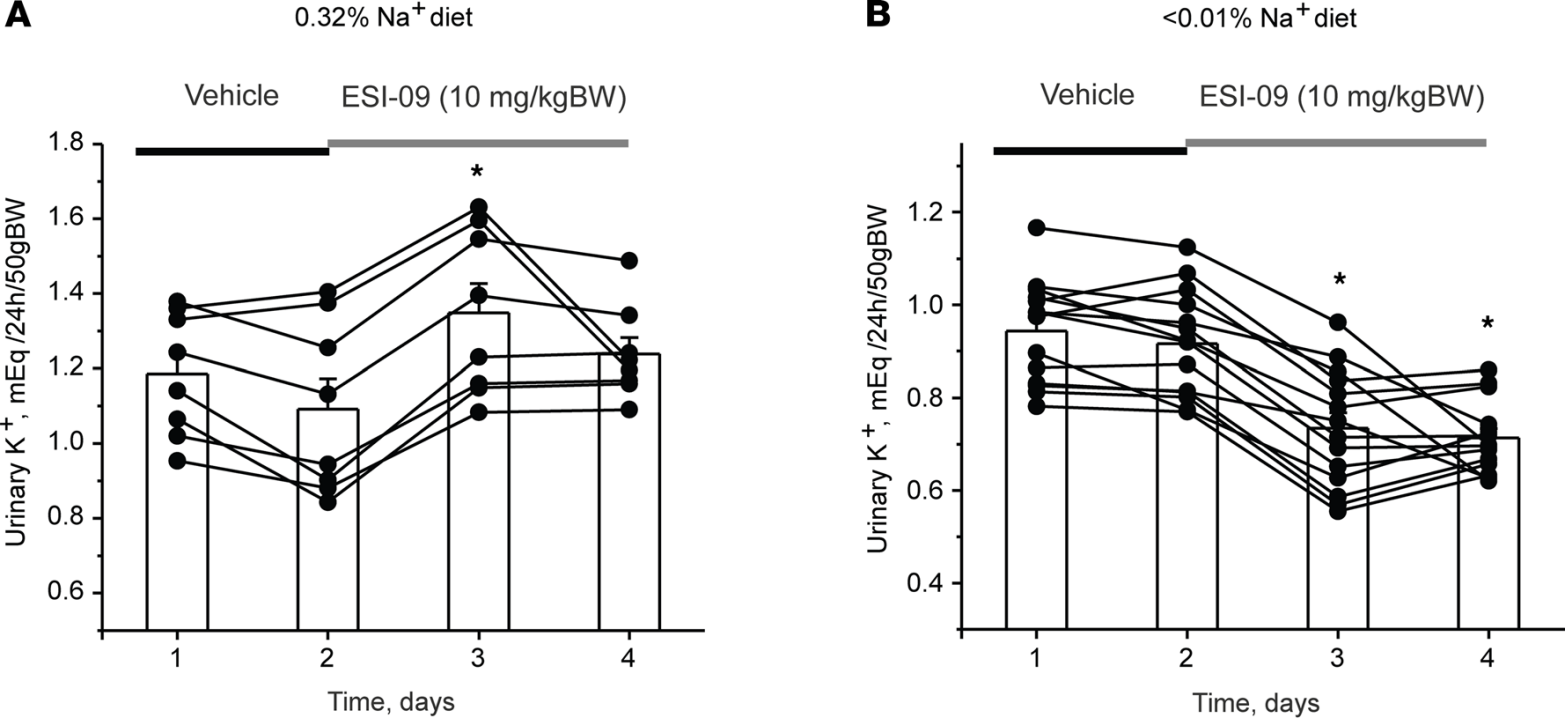
Opposite effects of Epac blockade with ESI-09 on urinary levels of K^+^ in mice kept on regular and sodium-deficient diets. The summary graphs show a time course of changes in 24-hour urinary K^+^ levels in Epac WT mice kept on (**A**) regular (0.32% Na^+^) and (**B**) sodium-deficient (< 0.01% Na^+^) diets upon daily injections of the combined Epac1&2 blocker ESI-09 (10 mg/kgBW) or vehicle as indicated with black and gray bars, respectively. Connected dots represent consecutive measurements from the same animal. *P < 0.05 versus vehicle; 1-way repeated ANOVA with post hoc Bonferroni test was used.

We previously reported that deletion of Epac1 and Epac2 significantly reduced NHE-3 levels in the proximal tubule (30). Thus, we next monitored changes in K^+^ levels in urine to establish the relative contribution of the Na^+^ reabsorption inhibition in the proximal tubule (increases urinary levels of K^+^) and collecting duct (decreases urinary levels of K^+^) to the observed natriuretic effects of systemic Epac inhibition with ESI-09. As summarized in Figure 9A, ESI-09 injection moderately increased K^+^ excretion in mice kept on a regular (0.32% Na^+^) diet. In contrast, urinary K^+^ levels were significantly decreased in all mice kept on a sodium-deficient (< 0.01% Na^+^) diet (Figure 9B). Injection of vehicle did not affect K^+^ levels on either the regular or sodium-deficient diet (Supplemental Figure 4). Overall, our results in Figures 8 and 9 suggest that the modest natriuretic effect of ESI-09 is determined by its effects in the proximal tubule of mice kept on the regular Na^+^ intake, whereas inhibition of ENaC-mediated Na^+^ reabsorption became central during dietary Na^+^ deficiency.

## Discussion

**Figure 10:**
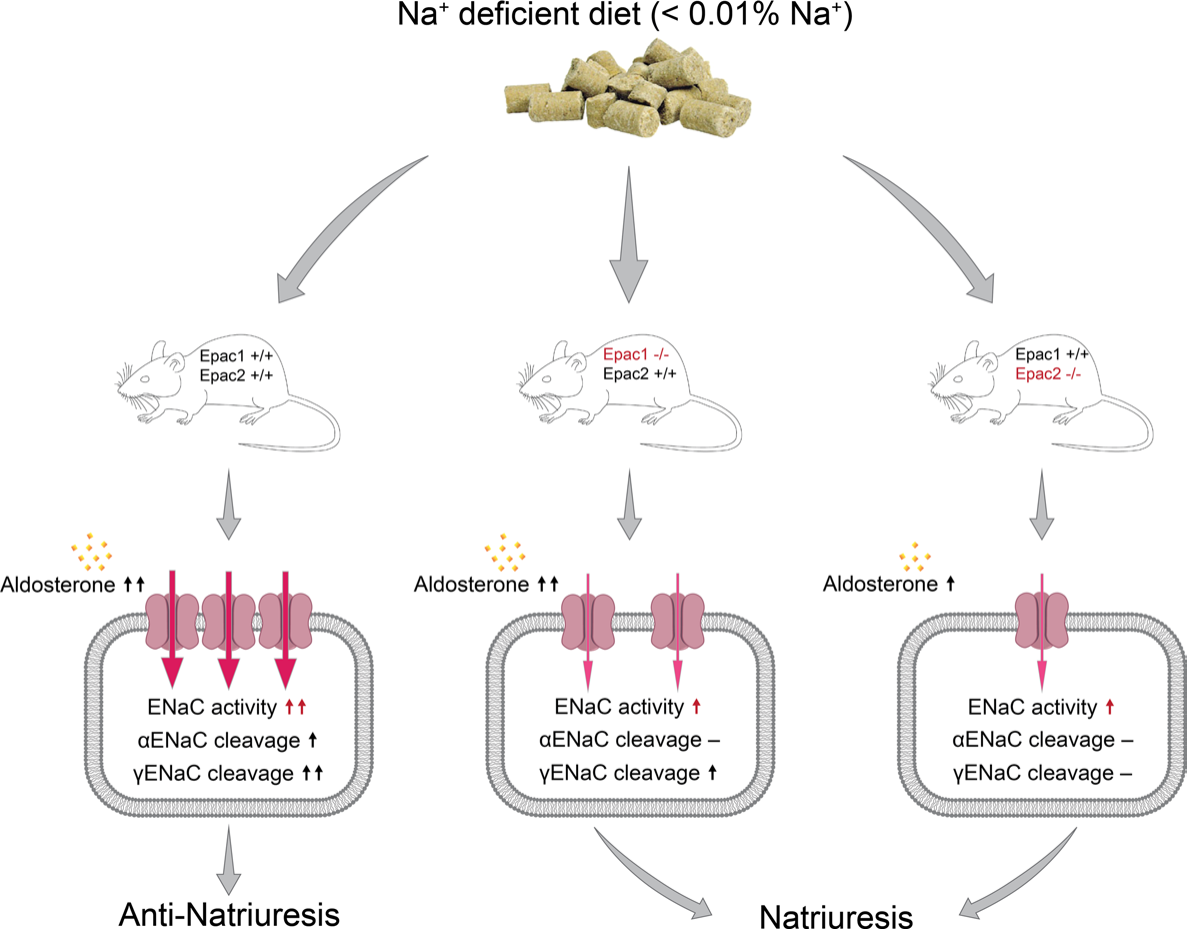
Principal scheme outlining regulation of ENaC activity and expression in the collecting duct by dietary sodium restriction in Epac WT, Epac1^–/–^, and Epac2^–/–^ mice.

In this manuscript, we demonstrate a critical role of Epac signaling cascade in the regulation of renal sodium handling and specifically ENaC activity in the collecting duct. We report a nonredundant contribution of Epac1 and Epac2 isoforms in stimulation of ENaC activity and expression in response to dietary sodium restriction (Figure 10). Mice lacking Epac1 exhibit a defective anti-natriuretic response largely due to decreased ENaC activity and compromised αENaC cleavage. Similarly, Epac2^–/–^ mice have much reduced upregulation of ENaC activity in response to dietary salt restriction and demonstrate disrupted cleavage of α and γENaC subunits. Simultaneous deletion of both Epac isoforms further mildly reduces ENaC activity despite compensatory upregulation of aldosterone levels. Finally, natriuretic response to systemic blockade of Epac cascade with ESI-09 is predominantly determined by inhibition of the ENaC-mediated Na^+^ reabsorption during dietary sodium restriction.

One of the major findings of this study is that genetic ablation or pharmacological inhibition of Epac signaling strongly inhibits ENaC activity on a sodium-deficient diet, whereas the effect is very mild in mice on the regular sodium regimen (Figures 6 and 7). Compelling evidence suggests a major role of the mineralocorticoid aldosterone in regulation of ENaC activity by dietary sodium restriction (3). In addition, an important complementary role of Ang II has also been convincingly demonstrated (10, 11, 35). Thus, it is reasonable to assume that aldosterone and/or Ang II signals to ENaC largely in an Epac-dependent manner. While the exact molecular details are currently not known, one possibility is that both aldosterone and Ang II could increase cAMP levels in the collecting duct cells (13, 14), which, in turn, stimulate its direct downstream effector, Epac. In addition, aldosterone has been shown to activate a monomeric G-protein, K-Ras, to increase ENaC activity and open probability via phosphoinositide 3-OH kinase (PI3-K) and augmented PI (3,4,^5^) P_3_ levels (36–38). Importantly, Epac serves as a guanosine exchange factor for small monomeric G-proteins Rap1 and Rap2 (20), which also belong to the Ras family. It should also be noted that cAMP has been recognized to control vesicular ENaC trafficking to the apical membrane (16, 39), and this mechanism is greatly potentiated in the presence of aldosterone (17). Overall, the available correlative evidence supports the idea that Epac cascade is well-positioned to increase ENaC activity and plasma membrane levels in response to aldosterone. This is consistent with marked reductions of ENaC activity in mice lacking Epac isoforms despite compensatory elevation of aldosterone (Figure 1B). Future studies are necessary to tackle the molecular details of the intermediate signaling mechanisms coupling aldosterone, Epac, and ENaC activity in the collecting duct principal cells.

We show that both Epac1 and Epac2 contribute to stimulation of ENaC activity and posttranslational processing by dietary salt restriction (Figures 3–5). Despite apparent structural similarities and molecular pathways for downstream signal production, Epac1 and Epac2 are not interchangeable, since deletion of either isoform produced notable reductions of ENaC activity and augmented urinary Na^+^ excretion on a low sodium diet. One would expect that the concomitant deletion of both isoforms could be cumulative with respect to natriuresis and decreased ENaC activity. However, our results (Figures 1–3) show only a marginally stronger phenotype compared with that in Epac1^–/–^ and Epac2^–/–^ mice. At the same time, we observed a much stronger compensatory elevation of aldosterone in Epac1&2^–/–^ (Figure 1B), which could mitigate the cumulative effect of Epac1&2 deletion. It should be noted, though, that single-channel ENaC activity was modestly lower in Epac1&2^–/–^ mice compared with Epac1^–/–^ and Epac2^–/–^, despite roughly 2-fold higher aldosterone levels in the former (Figure 3). Another possibility is that Epac1 and Epac2 might be critical regulators of sequential steps of the same single pathway of ENaC stimulation by aldosterone. Consistently, it was previously shown that subcellular localization of Epac1 and Epac2 overlap only partially in renal tubule, including the collecting duct cells (29). Vigorous studies show that Epac1 and Epac2 form discrete multimolecular signaling complexes within a cell, allowing isoform-specific control of various signaling pathways or different stages of the same pathway with minimal redundancy and compensation (40–42). In this case, deletion of either isoform would produce a comparable but not identical Na^+^-wasting phenotype with double KO producing a marginally worse phenotype, as is observed in the current study. In agreement with this notion, we showed that a pan-Epac1&2 blocker, ESI-09, produced a slightly stronger inhibitory effect on ENaC activity in split-opened collecting ducts than ESI-05, a selective Epac2 blocker (Figure 6). Moreover, ESI-09 had a mild but significant inhibitory effect on ENaC activity in Epac1^–/–^ and Epac2^–/–^ mice (Figure 7) most likely by targeting the intact counterpart isoform. It is also worth mentioning that Epac signaling to ENaC exhibits a certain degree of specificity, since deletion of either Epac1 or Epac2 did not interfere with AVP-dependent regulation of AQP2 trafficking to the apical plasma membrane of the collecting duct cells (30).

While our results demonstrate the direct effect of acute Epac inhibition on ENaC (Figure 6), the potential contribution of distorted extrarenal factors in mice with global deletion of Epac1 and Epac2 cannot be excluded. Particularly, cAMP–Epac rather than cAMP–PKA signaling has been shown to regulate aldosterone secretion in *zona glomeruloza* cells of the adrenal cortex (43). Of note, Epac2 expression is higher than Epac1 in the adrenal gland (44). This would be consistent with moderately lower aldosterone levels in Epac2^–/–^ than in Epac1^–/–^ mice (Figure 1B), which, in turn, contributes to a mildly greater natriuresis in the former in response to dietary sodium restriction (Figure 1A). In contrast, another study reported that stimulation of the cAMP–Epac pathway with 8-pCPT-2’-O-Me-cAMP had no effect on aldosterone production by adrenal gland (45). Regardless, we do not anticipate that extrarenal factors play a major role in the observed natriuresis. Thus, mice with double Epac isoform deletion exhibit an augmented aldosterone response to dietary Na^+^ restriction (Figure 1B). At the same time, ENaC activity was markedly decreased in Epac1&2^–/–^ compared with WT and mildly reduced compared with single Epac KOs (Figure 3). Overall, this suggests that the disturbed Na^+^ balance originates largely from defective Na^+^ reabsorption in the kidney. Additional studies using animal models with collecting duct-specific ablation of Epac should sort out the relative contributions of the direct and extrarenal factors in controlling ENaC activity during variations of dietary sodium intake.

Our results provide proof-of-principle evidence that Epac inhibitors could be used as natriuretics in the clinical setting. The important aspect is that Epac blockers only marginally inhibit ENaC activity during regular salt intake (Figure 6, E–G), whereas they effectively impede upregulation of ENaC activity (Figure 6, A–D) even in the presence of elevated aldosterone. Consistently, systemic administration of Epac blocker ESI-09 produces a mild natriuresis and kaliuresis on the regular Na^+^ intake, indicative of the predominant contribution of the proximal tubule (Figures 8 and 9). In contrast, ESI-09 produces much greater relative natriuresis and, importantly, anti-kaliuresis under dietary Na^+^ restriction, pointing to the dominant contribution of the collecting duct. Thus, inhibition of Epac cascade might be most effective during pathological states, which are associated with inappropriately high ENaC activity, for instance, in low renin and/or salt-sensitive hypertension (7, 46, 47). Second, Epac inhibition does not block ENaC completely, which, in conjunction with increased fluid delivery to the collecting duct due to diminished Na^+^ reabsorption in the proximal tubule (30), should not interfere too much with urinary excretion of K^+^, thus precluding the development of life-threatening hyperkalemia. Indeed, we did not detect major alterations in plasma K^+^ levels in Epac1^–/–^ and Epac2^–/–^ mice on either the regular or Na^+^-deficient diet (Supplemental Figure 1). Thus, Epac inhibitors would not likely cause hyperkalemia, as is commonly observed when classical ENaC pore blockers, such as amiloride, are used. Third, upregulation of Epac signaling has been noted in some pathological states, including cancer, diabetes, and polycystic kidney disease (48, 49). It is quite possible that hypertension, which is a common complication during these disorders, might be in part associated with ENaC overactivation and can be ameliorated with Epac inhibitors, such as ESI-09. However, careful and long-term future studies are necessary to address the efficiency and safety of Epac inhibition in treatment of hypertensive states.

### Perspectives.

This manuscript provides direct multicomponent evidence in support of regulation of ENaC activity in the renal collecting duct by Epac signaling during variations in dietary Na^+^ intake. We show that the deletion of Epac1 and Epac2 impairs stimulation of ENaC and compromises renal conservation of sodium in response to dietary sodium deficiency. We further demonstrate that systemic Epac inhibition leads to natriuresis, which is mainly determined by inhibition of ENaC activity. We speculate that Epac inhibition might be beneficial to block inappropriately high ENaC activity, as it commonly occurs in various hypertensive states.

## Methods

### Reagents and animals.

All chemicals and materials were from Sigma, VWR, Thermo Fisher Scientific, and Tocris unless otherwise noted and were at least of reagent grade. For experiments, 6- to 10-week-old Epac WT (C57Bl/6), Epac1^–/–^, and Epac2^–/–^ male mice were used. Usage of mice lacking Epac isoforms has been described (30, 50, 51). It was also demonstrated that deletion of either Epac isoforms does not result in a compensatory increase of its counterpart (23). Mice with concomitant deletion of both Epac isoforms (Epac1&2^–/–^) were created by sequential cross-breeding of Epac1^–/–^, Epac2^–/–^, and their heterozygous progeny. The genotyping primers were 5′-CTGGCCTCTCCTGAATCTTG and 5′-CCTCGCTGTTGGTAAGTGGT (for detection of WT Epac1 allele at 267 bp); 5′-AATGGGCTGACCGCTTCCTCGT and 5′-GCCATAGCCTCAACAAGCTC (for detection of Epac1^–/–^ allele at 500 bp); 5′-CCTCCCTTTTGCTCTCTCCT and 5′-CGCTCGCTGCATTTGTATTA (for detection of WT Epac2 allele at 350 bp); and 5′-AATGGGCTGACCGCTTCCTCGT and 5′-CGCTCGCTGCATTTGTATTA (for detection of Epac2^–/–^ allele at 300 bp). Supplemental Figure 2 shows representative PCR images of genotyping of Epac1^–/–^, Epac2^–/–^, and Epac1&2^–/–^, respectively.

### Systemic measurements.

Mice were acclimated for 3 days in metabolic cages (Techniplast, 3600M021) with free access to water and regular rodent chow (0.32% Na^+^; Envigo,TD.7012). Following acclimation, 24-hour urine samples were collected and assessed. As necessary, mice were fed with a Na^+^-deficient diet (< 0.01% Na^+^; Envigo, TD.90228) for at least 2 days or longer (up to 7 days) as described in the experimental protocols. Urinary creatinine concentration was assessed with QuantiChrom Creatinine Assay Kit (BioAssay Systems, DICT-500) utilizing improved Jaffe method (52). Aldosterone was measured using an enzymatic immunoassay kit (Cayman Chemical, 501090) in accordance with the vendor’s protocol. Urinary and plasma Na^+^ and K^+^ concentrations were measured using Jenway PFP7 Flame photometer (Bibby Scientific).

ENaC-induced natriuresis and anti-kaliuresis were assessed as the difference in respective urinary Na^+^ and K^+^ levels in sodium restricted Epac WT, Epac1^–/–^, Epac2^–/–^, and Epac1&2^–/–^ mice for 6 hours before and following i.p. injection of amiloride (2 mg/kgBW). For i.p. injections of Epac1&2 inhibitor in mice, ESI-09 was freshly prepared in sterile 10% ethanol/Tween 80 and 90% PBS in concentration of 10 mg/mL and the injection volume was proportional to the animal weight.

### Western blotting.

Immediately after dissection, kidneys were placed on ice, decapsulated, and homogenized in 3 volumes of ice-cold lysis buffer containing 50 mM TrisCl, 5 mM EDTA and 1% Triton X-100 (pH 7.5) supplemented with Complete Mini protease and PhosSTOP phosphatase inhibitor cocktails (Roche Diagnostics). The homogenates were centrifuged at 1000 *g* for 15 minutes at +4°C and sediment was discarded. Protein concentration was determined with a Bradford assay using BSA as a standard. The samples (40 μg/lane) were separated on 9% polyacrylamide gels at 150 V for 90 minutes and transferred to a nitrocellulose membrane for 1.5 hours at 100 V. Equal protein load was verified by Ponceau red staining using standard procedures. Nitrocellulose membranes were incubated with primary anti-αENaC antibodies (rabbit polyclonal, 1:1000; Stress Marq Biosciences, SPC-403) or anti-γENaC antibodies (rabbit polyclonal, 1:1000; Stress Marq Biosciences, SPC-405) overnight at +4°C. Upon washout (3 times for 10 minutes in TBS-Tween), the membrane was incubated with peroxidase-conjugated goat anti-rabbit (1:10000; Jackson ImmunoResearch Laboratories) secondary antibodies for 1 hour at room temperature. Blots were quantified using ImageJ 1.50 software (NIH). The intensities of the studied protein bands were normalized to the total signal of the respective line in Ponceau red staining.

### Isolation of individual collecting ducts.

The procedure for isolation of the collecting ducts from Epac WT, Epac1^–/–^, Epac2^–/–^, and Epac1&2^–/–^ mice suitable for patch clamp electrophysiology closely followed the protocols previously published by our group (53, 54). Briefly, kidneys were cut into thin slices (< 1 mm) with slices placed into an ice-cold solution that contained (in mM): 150 NaCl, 5 KCl, 1 CaCl_2_, 2 MgCl_2_, 5 glucose, and 10 HEPES (pH 7.35). Collecting ducts were visually identified by their morphological features (pale color, coarse surface and, in some cases, bifurcations) and were mechanically isolated from kidney slices by microdissection using watchmaker forceps under a stereomicroscope. Isolated collecting ducts were attached to 5 × 5 mm cover glasses coated with poly-L-lysine. A cover-glass containing a collecting duct was placed in a perfusion chamber mounted on an inverted Nikon Eclipse Ti microscope and perfused with a bath solution at room temperature. Collecting ducts were split-opened with 2 sharpened micropipettes, controlled with different micromanipulators, to gain access to the apical membrane. As necessary for experimental design, ESI-05 (5 μM), ESI-09 (5 μM), or vehicle (DMSO; 1:1000) was applied to the perfusion chamber during split-opening. The tissues were used within 2 hours of isolation.

### Single channel ENaC recordings in split-opened collecting ducts.

ENaC activity was determined in cell-attached patches on the apical membrane made under voltage-clamp conditions (−*V_p_* = −60 mV) using standard procedures (10, 11, 52). Current recordings were made in a permanently perfused bath (1.5 mL/min). Recording pipettes had resistances of 8–10 megaOhms. Typical bath and pipette solutions were (in mM): 150 NaCl, 5 KCl, 1 CaCl_2_, 2 MgCl_2_, 5 glucose and 10 HEPES (pH 7.35); and 140 LiCl, 2 MgCl_2_ and 10 HEPES (pH 7.35), respectively. Gap-free single-channel current data from gigaOhm seals were acquired and analyzed with Axopatch 200B (Molecular Devices) patch clamp amplifier interfaced via a Digidata 1440 (Molecular Devices) to a PC running the pClamp 10.5 suite of software (Molecular Devices). Currents were low-pass filtered at 100 Hz with an eight-pole Bessel filter (Warner Instruments). Events were inspected visually prior to acceptance. ENaC activity was analyzed over a span of 60–120 seconds for each experimental condition. Using previously described analysis (11), we can reliably (*P* < 0.05) estimate the maximal number of functional ENaC in a patch using this time span. Channel activity in individual patches, defined as *NP*_o_, was calculated using the following equation: *NP_o_* = (*t*_1_ + 2*t*_2_ + … + *nt_n_*), where *N* and *P*_o_ are the number of ENaC in a patch and the mean open probability of these channels, respectively, and *t_n_* is the fractional open time spent at each of the observed current levels. *P*_o_ was calculated by dividing *NP_o_* by the maximal number of simultaneously active channels within a patch (*N*) as defined by all-point amplitude histograms. To estimate total ENaC activity (*fNP_o_*) in a particular experimental group, we normalized *NP_o_* to the frequency of observing patches with at least 1 active channel (*f* = number of patches with active channels/total number of patches). To assess functional ENaC expression for each experimental condition, the mean of number of active channels (*N*) within a patch was also normalized by *f*. For representation, current traces were adjusted for a slow baseline drift as necessary.

### Statistics.

All summarized data are reported as mean ± SEM. Statistical comparisons were made using 1-way ANOVA with a post hoc Tukey test or 1-way repeated ANOVA with Bonferroni’s post hoc test (for paired experiments within the same group). A *P* value of less than 0.05 was considered significant.

### Study approval.

Animal use and welfare adhered to the NIH *Guide for the Care and Use of Laboratory Animals* (National Academies Press, 2011) following protocols reviewed and approved by the Animal Care and Use Committee of the University of Texas Health Science Center at Houston.

## Author contributions

The research studies were designed by OP, A Staruschenko, and XC. VNT, KP, A Stavniichuk, NHK, GR, OZ, FCM, SK, and OP conducted the experiments and analyzed the data. FCM and XC provided the materials. OP wrote the manuscript.

## Supplementary Material

Supplemental data

## Version 1 12/16/2021

In-Press Preview

## Version 2 02/08/2022

Electronic publication
