# Supplementary material for: Epac1–/– and Epac2–/– mice exhibit deficient epithelial Na+ channel regulation and impaired urinary Na+ conservation
Source: JCI Insight. 2022 Feb 8;7(3):e145653. doi: 10.1172/jci.insight.145653 (PMC8855822; doi:10.1172/jci.insight.145653)
Supplement: Supplemental data [file jciinsight-7-145653-s057.pdf]

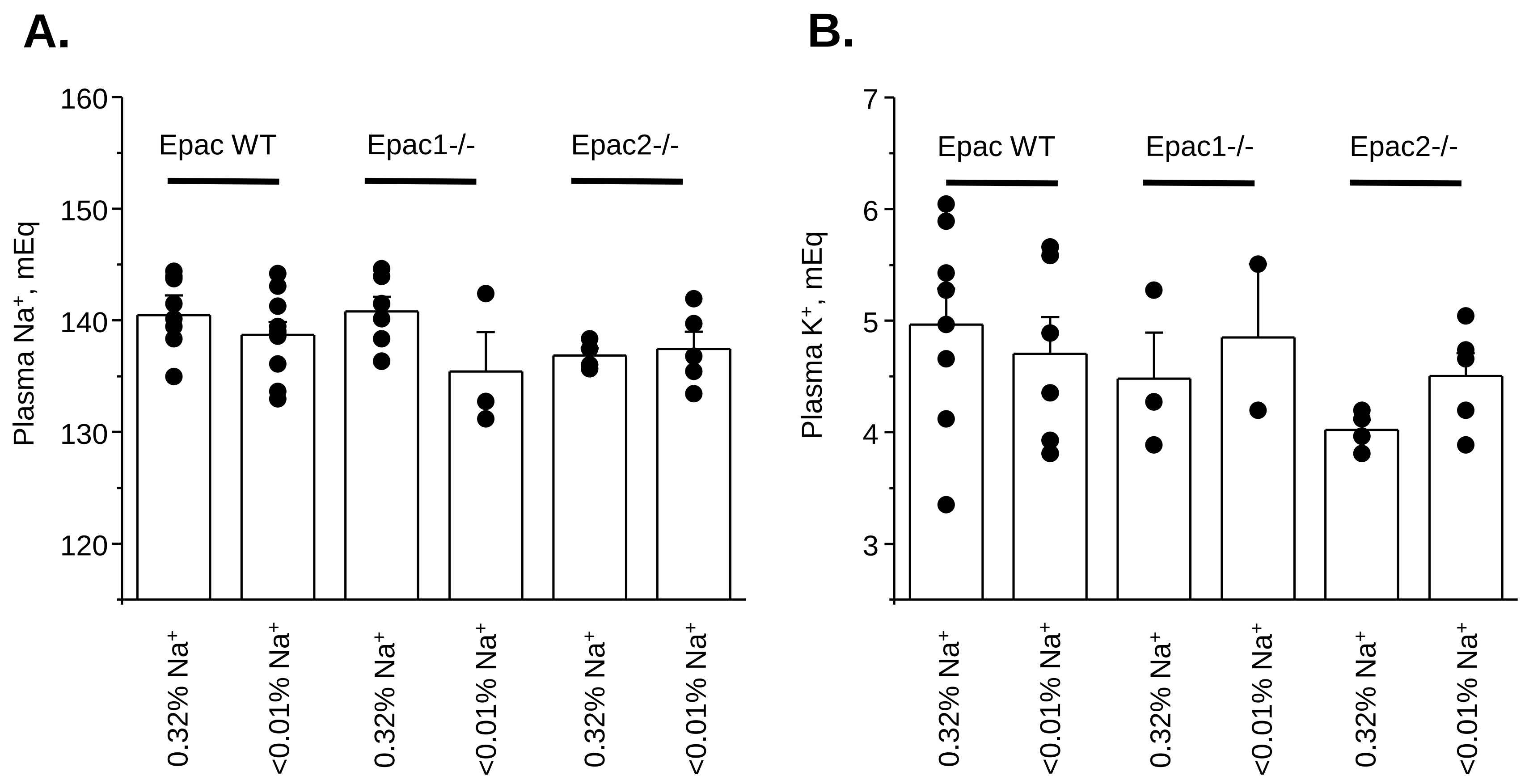

Figure S1. Deletion of Epac isoforms does not affect plasma concentration of Na^+ and K^+. The summary graphs showing a comparison of plasma Na^+ **(A)** and K^+ **(B)** levels in Epac WT, Epac1 -/-, and Epac2 -/- mice kept on regular (0.32% Na^+) and sodium deficient (<0.01% Na^+) diets. Individual measurements from different animals are shown with dots.

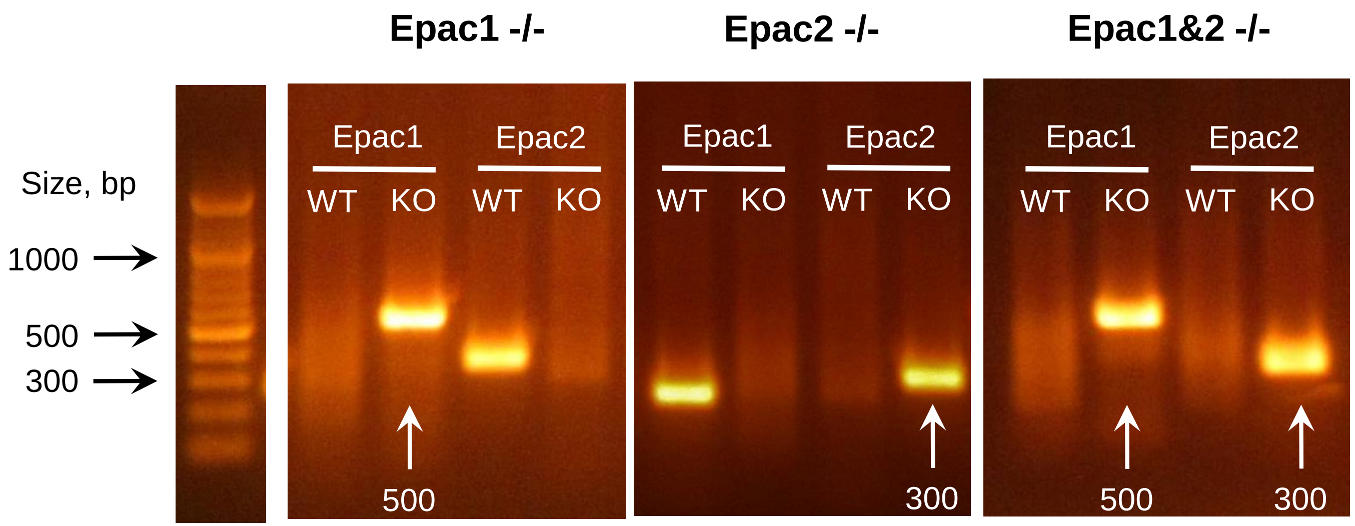

<u>Figure S2. Creation of Epac1&2 -/- mice.</u> Representative genotyping PCRs for Epac1 -/-, Epac2 -/-, and Epac1&2 -/- probing for the respective wild type (WT) and knockout (KO) allele of genes encoding Epac1 and Epac2. The anticipated size of respective PCR product is shown for KO alleles.

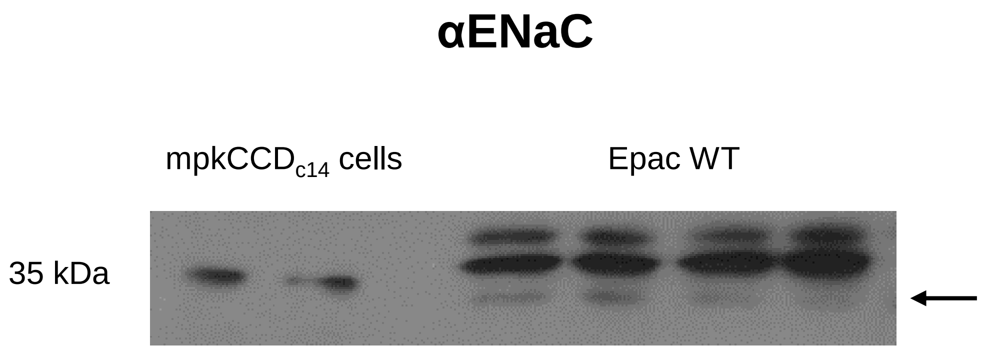

Figure S3. Comparison of chemiluminescent signal reporting cleaved αENaC subunits from lysates of cultured mpkCCD$_{c14}$ cells (left) and whole kidney lysates of Epac WT mice kept on a regular (0.32% Na^+) diet. The presence of the common band around 35 kDa in both preparations is highlighted with an arrow.

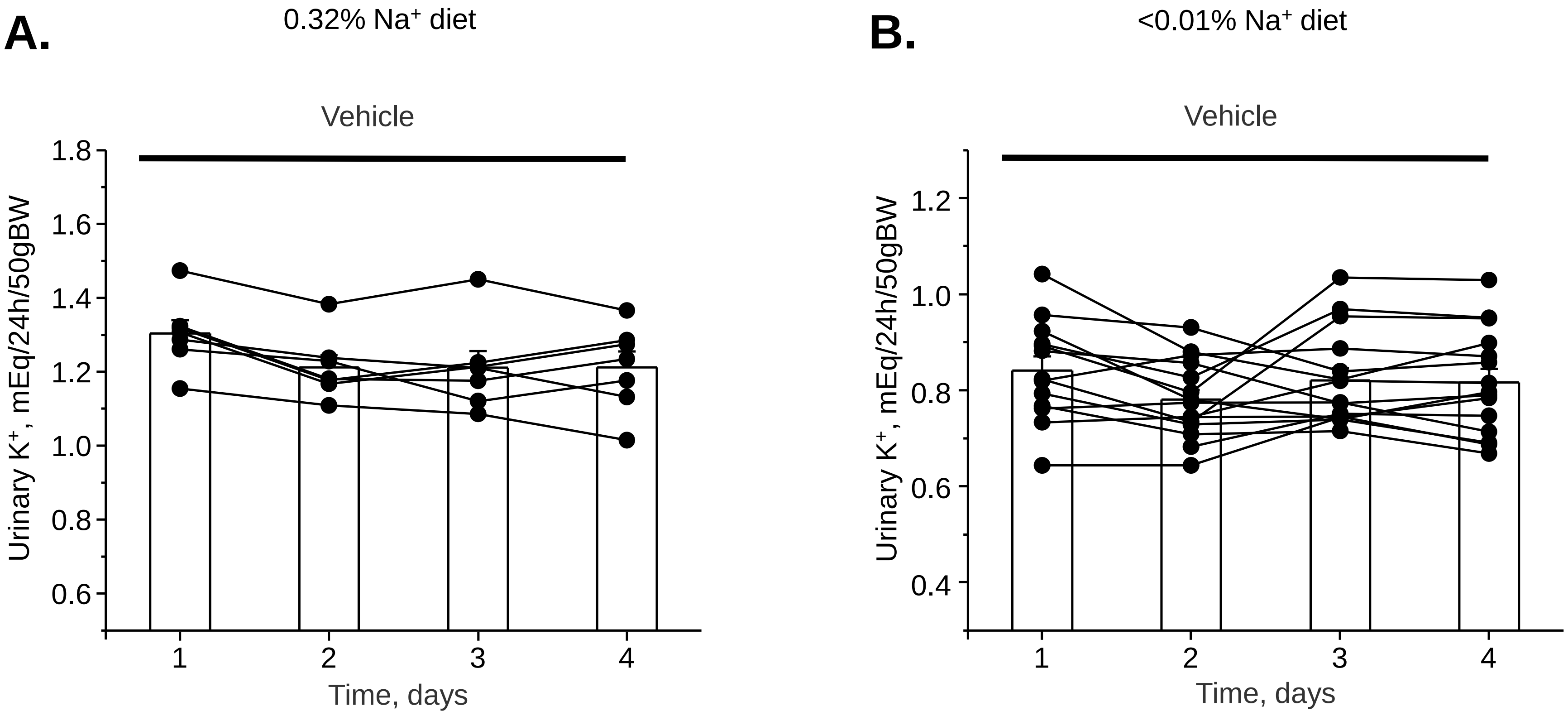

<u>Figure S4. Daily injections of vehicle for ESI-09 do not affect urinary levels of K^+.</u> The summary graphs showing a time course of changes in 24 h urinary K^+ levels in Epac WT mice kept or regular (0.32% Na^+) **(A)** and sodium deficient (< 0.01% Na^+) **(B)** diets upon daily injections of vehicle (sterile 10% ethanol/Tween80 and 90% phosphate buffer saline) for Epac1&2 blocker, ESI-09.
